# SARS-CoV-2 in companion animals: Do levels of SARS-CoV-2 seroconversion in pets correlate with those of pet’s owners and with protection against subsequent SARS-CoV-2 infection?

**DOI:** 10.1080/21505594.2022.2098922

**Published:** 2022-08-01

**Authors:** Hannah Murphy, Shania Sanchez, Shamim Ahmed, Md. Mizanur Rahman, Da Di, Mythili Dileepan, Daniel Heinrich, Yuying Liang, Hinh Ly

**Affiliations:** a Department of Veterinary & Biomedical Sciences, College of Veterinary Medicine, University of Minnesota, Twin Cities, MN, USA; b Department of Veterinary Clinical Sciences, College of Veterinary Medicine, University of Minnesota, Twin Cities, MN, USA

**Keywords:** SARS-CoV-2, seroprevalence, felines, canines, ELISA, Minnesota

Domestic cats (felines) and dogs (canines) live in close contact with hundreds of millions of people across the United States of America (USA) and around the globe. In the era of the COVID-19 pandemic, determining the prevalence and susceptibility of pet cats and dogs to SARS-CoV-2 infection is crucial as it can have a significant impact on public health [1–3]. While SARS-CoV-2 appears to have a zoonotic origin, it has also been shown to be transmitted from humans to various species of animals (i.e. reverse zoonosis) as summarized in our review article published recently in the Virulence journal [4]. There have been many reports showing evidence of SARS-CoV-2 transmission from human owners to their pets [1,5–8] however, it is not clear whether there is a direct correlation between infection levels of humans and their pets. Therefore, it is important to assess the degree of SARS-CoV-2 prevalence in susceptible pets (cats and dogs) and to determine whether there is a correlation between the level of infection in pets and their human owners.

In June 2021, we published an original research article in Virulence [9] in which we reported results of a serological survey of SARS-CoV-2 exposures in pet dogs and cats that were brought to the Veterinary Medical Center (VMC) at the University of Minnesota, Twin Cities for routine check-ups or for other clinical diagnoses unrelated to COVID-19. Discarded serum samples of 239 pet cats and 510 pet dogs were collected during the early phase of the COVID-19 epidemic in Minnesota between mid-April and mid-June 2020. To determine the seroprevalence of SARS-CoV-2 antibodies in those companion animals, we developed enzyme-linked immunosorbent assays (ELISAs) using the recombinant SARS-CoV-2 nucleocapsid (N) protein and the receptor-binding domain (RBD) of the spike protein as the antigens to capture and quantify the amount of the respective antibodies that might be present in serum samples of the Minnesotan pets. Our data showed that, during this early phase of the COVID-19 epidemic in the USA (i.e., spring of 2020), the average seroprevalence of N- and RBD-specific antibodies in pet cats was 7.9% (Figure 1A) and 2.9% (data not shown here but was previously reported in our original research article [9]), respectively. On the contrary, pet dogs were found to have a very low percentage of SARS-CoV-2 specific antibodies (~1%), regardless of the type of ELISAs used [9] (Figure 1A).

**Figure 1.**
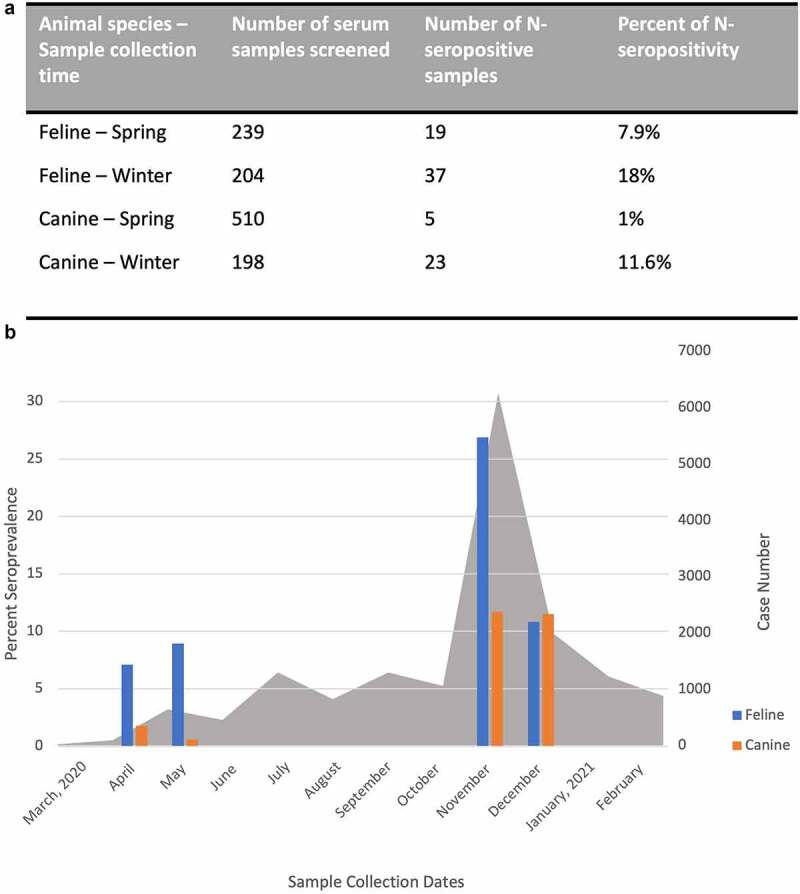
a) Feline and canine serum samples were collected during the spring (from mid-April to mid-June 2020) and winter months (from mid-November 2020 to mid-January 2021) in Minnesota, USA, and were used to screen for serological evidence of SARS-CoV-2 exposure by the SARS-CoV-2 *N*-based enzyme-linked immunosorbent assay (ELISA). b) Graph comparing the levels of SARS-CoV-2 seropositivity in feline (blue bars) and canine (orange bars) serum samples in the spring and winter months when there is a surge in the number of new daily cases of COVID-19 in humans in Minnesota (grey shaded areas). Data for human daily COVID-19 cases was reported [10]. Percent seroprevalence of SARS-CoV-2 in feline and canine species are shown on the left Y-axis and the human daily COVID-19 case number are shown on the right Y-axis over time(x-axis).

During the later (winter) months of 2020, there was a surge in confirmed human daily COVID-19 cases and associated hospitalizations and deaths in Minnesota [11] (Figure 1B). As companion animals might be living in even closer quarters to their human owners during the winter months than in the spring, and therefore were more likely to be susceptible to SARS-CoV-2 due to reverse zoonotic transmission events, we hypothesized that the seroprevalence of companion animals could correspondingly be increased during the winter months of 2020 in Minnesota. Using available archival (discarded) serum samples collected from pet cats and dogs that were also brought to the VMC of the University of Minnesota from mid-November 2020 to mid-January 2021, we screened 204 serum samples of pet cats and 198 serum samples of pet dogs for seroprevalence of SARS-CoV-2. We used the same N-based ELISA as described in our previous report [9] for screening purposes and found 18% (37/204) of N-specific antibodies in pet cats and 11.6% (23/198) in pet dogs during these winter months in the state of Minnesota (Figure 1A). Evidently, significantly higher numbers of companion animals living in Minnesotan households during the winter COVID-19 surge in the human population were found to be seroconverted than during the early phase of the COVD-19 epidemic (Figure 1B). Noticeably, the increases in the rate of pet’s COVID-19 seroprevalence appeared to correspond with the surge in human daily cases of COVID-19 during the winter months [10,11].

The state of Minnesota is not the only place where the increased seroprevalence correlation appears to be true. In a COVID-19 One Health household transmission investigation in the states of Utah and Wisconsin from April to May 2020, more seropositive pets were found in households with a greater rate of human household secondary transmissions [5], which were defined as family members (or people living in the same living space) who became infected by the primary-infected individual within one’s incubation period of COVID-19 symptom onset. Among 47 pets tested, serological results showed that 17% (4/37 dogs and 4/19 cats) had detectable SARS-CoV-2 neutralizing antibodies (nAbs). Of note, the authors found similar rates of SARS-CoV-2 infection among pet cats (21% or 4/19 by RT-PCR and sequencing) in this study as reported in another study in Texas (17.6% or 3/17 cats by RT-PCR and sequencing), where pet cat and dog samples were collected from the end of June to the end of July 2020 [12,13]. However, initial sampling showed higher rates of infection, e.g., 43.8% (7/16) of cats and 11.9% (7/59) of dogs had anti-SARS-CoV-2 nAbs in their sera that were either relatively stable or with increasing titres over the 2–3 months of follow up with no additional evidence of seroconversion. It is noteworthy that similarly high levels of SARS-CoV-2 infection among pet cats in Utah, Wisconsin, and Texas were found during the surge of human COVID-19 cases [14] as what we are reporting in the current study among pet cats during the human’s daily COVID-19 surge in Minnesota [10,11].

Correlative levels of SARS-CoV-2 infection in human owners and their pets also appear to be taking place outside the United States. Similar high rates of SARS-CoV-2 infections of pet cats (40%, 4/10 cats) and dogs (31%, 9/29 dogs) in 47.6% of tested households were found to be positive for SARS-CoV-2 by either RT-PCR or serology in Rio de Janeiro, Brazil between May and October of 2020 [6]. Neutralizing antibodies against SARS-CoV-2 were found in 3.4% of pet dogs (1/29) and 20% (2/10) of pet cats. A longitudinal approach was also used to study the transmission dynamics of SARS-CoV-2 as determined by RT-PCR and/or sequencing analysis. By using a serial sampling method, the authors were able to show that 41.7% (5/12) of the companion animals (pet cats and dogs of COVID-19 positive human owners) were positive for SARS-CoV-2 RNA during the second and third at-home sample collection visits [6]. Interestingly, the same authors found, using logistic regression analysis, that pet’s being neutered and sharing a bed with the infected human owners were more likely to be associated with pet’s SARS-CoV-2 transmission, which supports the idea that increased proximity to pets when the human pet’s owners are sick can increase the likelihood of SARS-CoV-2 transmission to companion animals [6]. This finding was bolstered by a related study, which found that households where the index COVID-19 patient decreased their interaction with pets after their COVID-19 diagnosis had zero pet’s transmission (as determined serologically), whereas COVID-19 human patients with increased duration of their interactions with pets had a higher chance of transmitting the virus to their pets [13].

A study in Italy [7], which looked at the evidence of exposure to SARS-CoV-2 in cats and dogs from COVID-19 positive households, sampled 603 pet dogs and 316 pet cats from different Italian regions which were severely impacted by COVID-19 outbreaks. Overall, they detected nAbs in the serum of 15 of 451 dogs (3.3%) and 11 of 191 cats (5.8%) as determined by plaque reduction neutralization test (PRNT) [7]. Among samples from households with known human COVID-19 status, 6/47 dogs and 1/22 cats had nAbs in their sera [7]. Interestingly, they found that dogs were significantly more likely to test positive for SARS-CoV-2’s nAbs if they came from a known COVID-19 positive household or were male [7]. When the authors looked at the Lombardy region, a region in Italy with a particularly high human COVID-19 disease outbreak, they found evidence that supports our hypothesis about a positive correlation between pet-associated SARS-CoV-2 seroprevalence and human’s COVID-19. Specifically, there was a positive correlation between the proportion of dogs that tested positive for SARS-CoV-2’s nAbs and the recorded COVID-19 disease burden in humans [7]. When those household pets were tested by the polymerase chain reaction (PCR) method, they were found to be negative for SARS-CoV-2, which appeared to contradict serology results. A potential explanation that the authors provided was that although pets could seroconvert, they were only shedding virus for a relatively short period of time [7]. This finding suggests that the longevity of the serological response in pet cats and dogs may potentially play a role in the correlation in seroprevalence between pets and their human owners.

Cats are considered one of the most susceptible animal species to SARS-CoV-2 infection [15] and although most experimentally and naturally infected cats are asymptomatic to mildly symptomatic, there are some cases where cats display a mild-to-severe respiratory syndrome [15–18]. Several studies have also shown that under these natural infection conditions, cats most often develop antibodies against SARS-CoV-2, but the duration of seropositivity seems to vary between the infected animals [6,19–22]. Even though most studies do not follow the same animals over time but rather different groups of animals at different time points to monitor potential long-term seropositivity, for the few studies that have looked at long-term antibody persistence, the duration of seroprevalence ranges from about four months, when the levels of binding and nAbs decrease to below the detection limit after 110 days after testing positive for SARS-CoV-2 antibodies by RBD-based ELISA [20] to ten months, when the levels of binding and nAbs are still detectable [19,21] for cats. Similarly, data reported in the Decaro study [19] showed that dogs appeared to have a similar range of antibody persistence (as assessed by detectable levels of binding and nAbs) that ranges from three to ten months. Collectively, these studies support the notion that pet cats and dogs can develop long-term binding and nAb responses against SARS-CoV-2 under natural infection conditions [19,23,24].

As previously mentioned, pets can produce nAbs against SARS-CoV-2 infection, but nAb production does not necessarily confer protection, as has been routinely observed in cases of human’s SARS-CoV-2 breakthrough infection. This is evidenced by examining the level of nAb titres across different human COVID-19 vaccine studies, in which a comparison of normalized nAb levels (i.e. neutralization titres were normalized to the mean convalescent titre using the same assay in the same study) and vaccine protective efficacy demonstrates a strong non-linear relationship between mean nAb level and reported protection level, thus suggesting (at least in humans) that nAb levels are highly predictive of immune protection from symptomatic SARS-CoV-2 infection [25]. Similarly, in experimentally infected cats, it appears that prior infection with SARS-CoV-2 can provide them with some levels of protection upon re-exposure to the virus, but it does not necessarily provide a level of sterilizing immunity [26–28]. Two separate studies by Chiba and colleagues [26] and by Bosco-Lauth and colleagues [27] found that cats were not reinfected upon re-exposure and could induce a significant level of neutralizing antibodies [26,27], meanwhile a study by Gaudreault and colleagues [28] found that cats could be re-infected but that virus shedding was in insufficient levels to spread the infection to naïve cohoused cats, thus, demonstrating a minimal or non-sterilizing level of immunity [28]. To the best of our knowledge, there does not appear to be any studies thus far that examine the potential levels of protective immunity against re-exposure to SARS-CoV-2 in dogs.

Taken all together, the aforementioned findings from around the world support the probable causes for the observable increases in seroprevalence for SARS-CoV-2 in pet cats and dogs, especially during the winter months, when there were significant increases in the number of daily human COVID-19 cases and of human-to-animal contacts, than during the spring time [9] (as an example, see Figure 1B). The levels of companion animal infections by SARS-CoV-2 appear to correspond with those levels observed in their human owners. Additionally, both pet cats and dogs appear to be able to develop some relatively long-term binding and nAb responses against SARS-CoV-2, which at least in cats, can provide some levels of protection against re-exposure, but they do not necessarily correlate with sterilizing immunity, as are often noted in human’s cases of SARS-CoV-2 breakthrough infection.

## Data Availability

Data reported in the figure and table in this article have been deposited in a recognized data repository (Figshare.com) with a digital object identifier (10.6084/m9.figshare. 19897738).
